# The Evolution of Sugar

**Published:** 1886-07

**Authors:** 


### THE EVOLUTION OF SUGAR.

Professor Falberg has made a great discovery, since which we need never feel that the civilized world will lack for any of its needs or luxuries. He has obtained from coal-tar a substance exceeding the sweetness of sugar by two hundred and thirty times. What this means for the world can only be fully appreciated by viewing it from an historical standpoint. In 1880 the inhabitants of Great Britain proved themselves, estimating the thing in matter-of-fact way, the sweetest people on earth ; they consumed in one year 18,374,543 cwt. of sugar, or 62.80 lbs. to each person in the kingdom. The United States came next, having used 13,040,500 cwt., or 37,80 lbs. for each individual.

Contrast this with the condition of the Greeks and Romans, who scarcely knew what sugar was. Even the ancient Hebrews had little or no knowledge of the sweet substance.

It is a question whether the world would have ever discovered that there was such a thing as sugar if it had not been for the conquests of the Great Alexander ; for it was Nearchus, Alexander's admiral, who found *saccharon* in the East indies, and brought back knowledge to the Greeks concerning it. It was Isodorus, 68 A. C., who again told the inhabitants of the Western world that there was "a fluid pressed from reeds sweeter than honey."

It was the great Galen who recognized the therapeutic value of sugar and first prescribed it, though before his day it had been spoken of and given its medicinal standing.

"If dissolved in water," said an ancient writer, "it is beneficial to the bowels and stomach." Behold the origin and warrant of the French use of *Eau Sucree :* "It is useful in diseases of the bladder and kidneys," he continues, "and when sprinkled in the eye removes those substances which obscure the sight."

It was thus that the world first cut its sweet tooth. How it has grown! Even a hundred years back sugar was a luxury common only with the rich ; now every person in the United States, every man, woman and child, averages nearly thirty-eight pounds of it a year.

Nine hundred patents have been taken out on the implements alone used in its manufacture. To obtain the sweet juices from the *Beta Vulgaris*, the *Saccharum Officinum*, the *Sorghum Saccharatum*, a most complicated process is employed.

And now the reader may be in a condition to contemplate what is meant by Prof. Falberg's discovery of a substance two hundred and thirty times as sweet as sugar, and made from coal-tar.

There has been much grumbling concerning the name of Saccharine which has been given to this new material, by those men who do not realize the difficulty in christening the newly-found coal tar sugar ; it does not sound any more appetizing than oleomargarine, and it would puzzle a child to demand over the grocer's counter benzoyl-sulphuric-nitrode, the name which the chemists give it. Hardly less startling are the initials of the above, or, more scientifically expressed, its formula :

$$\left( C_6 H_4 \lessdot {}^{Co}_{So} \gtrdot N H. \right)$$

Dr. Stutzer, of Bonn, has made some physiological experiments with it on dogs. Administered in large doses, it does not influence tissue change, and was perfectly non-injurious.

It has been used in Berlin hospitals for diabetic patients, instead of cane-sugar, without showing the disadvantages of the latter.

It is described as looking like flour, though denser, melts at 200° C., crystallises from an aqueous solution in thick, short prisms, soluble with difficulty in cold water—more readily in hot, as also in alcohol, ether, glucose and glycerine.

It is expensive at present, since the process of obtaining it is so complex, but as one ounce equals fourteen pounds of sugar, it is not so high as it would seem.—*Journal of Reconstructives.*.

